# Conduct disorder and hyperprolinemia type I: A case report

**DOI:** 10.1192/j.eurpsy.2023.482

**Published:** 2023-07-19

**Authors:** L. Olivier, Ó. De Juan, H. Andreu, L. Bueno, M. Llobet, A. Ortiz, A. Morer, L. Lázaro, D. Ilzarbe

**Affiliations:** ^1^Institut de Neurociènces, Hospital Clínic de Barcelona, Barcelona, Spain

## Abstract

**Introduction:**

Hyperprolinemia is defined by high proline levels of blood and its primary type consists on a metabolic disorder that is the result of a number of different genetic defects affecting the degradation of proline. The complex relationship between this disease and different psychiatric phenotypes has been an important subject of study in recent years, suggesting a “common psychiatric phenotype” (Namavar et al. Am J Med Genet B Neuropsychiatr Genet 2021; 186(5), 289-317), though its exact characteristics are yet to be determined. A higher prevalence of psychotic disorders (Guo et al. Metab Brain dis 2018; 33 89-97) explained through altered glutamate metabolism, autism spectrum disorders, developmental delay and intellectual disability has been proposed.

**Objectives:**

To describe the case of a patient, recently diagnosed of hyperprolinemia type I, presenting a conduct disorder alongside with ADHD, oppositional defiant disorder and an unspecified pervasive developmental disorder.

**Methods:**

We present the case of a 15-year-old male that has received follow-up care by our mental health services. The patient was born preterm (35+5 weeks) and required reanimation, oxygen therapy, antiretroviral therapy (biological mother was HIV positive) and pharmacological therapy with phenobarbital (in order to treat methadone withdrawal syndrome). It was adopted nationally when he was 18-month-old and experimented an adequate development during his first years, only highlighting slight psychomotor restlessness and distinctive facial features. During the next years, he receives diagnosis of ADHD (with little to no registered response to amphetamine derivatives), oppositional defiant disorder, social pragmatic communication disorder and fetal alcohol syndrome.

**Results:**

During his first hospital admission, a neuropediatrician was contacted to study the patient and recommended for a metabolic screening to be done, where high blood levels of proline were detected (940.1μmol/L). After this, a procedure of massive exome sequencing of genes that were known to be related to alterations in the metabolism of proline was conducted, finding the mutation c.[1357C>T] in the gen PRODH. This translates to an amino acid replacement in the protein proline dehydrogenase (p.[Arg453Cys]; [Arg453Cys]), which has been studied (Bender et al. Am J Hum Genet 2005; 76 409–420) that it reduced its activity in a 70%, making it a very probable cause of the hyperprolinemia.

**Conclusions:**

There is still scarce evidence of the psychiatric phenotypes presented in patients with hyperprolinemia. Further research is needed in order to accurately define the complex relationship between this metabolic disorder and its effect on the central nervous system.

**Disclosure of Interest:**

None Declared

